# *In situ* X-ray beam imaging using an off-axis magnifying coded aperture camera system

**DOI:** 10.1107/S0909049513011060

**Published:** 2013-05-18

**Authors:** Anton Kachatkou, Nicholas Kyele, Peter Scott, Roelof van Silfhout

**Affiliations:** a School of Electrical and Electronic Engineering, The University of Manchester, Sackville Street Building, Manchester M13 9PL, UK

**Keywords:** X-ray imaging, pinhole camera, scattering measurements, deconvolution, beam diagnostics

## Abstract

An imaging model and an image reconstruction algorithm for a transparent X-ray beam imaging and position measuring instrument are presented. The instrument relies on a coded aperture camera to record magnified images of the footprint of the incident beam on a thin foil placed in the beam at an oblique angle. The imaging model represents the instrument as a linear system whose impulse response takes into account the image blur owing to the finite thickness of the foil, the shape and size of camera’s aperture and detector’s point-spread function. The image reconstruction algorithm first removes the image blur using the modelled impulse response function and then corrects for geometrical distortions caused by the foil tilt. The performance of the image reconstruction algorithm was tested in experiments at synchrotron radiation beamlines. The results show that the proposed imaging system produces images of the X-ray beam cross section with a quality comparable with images obtained using X-ray cameras that are exposed to the direct beam.

## Abstract 2

This paper presents an imaging model and a reconstruction algorithm for obtaining X-ray beam cross-sectional images from the data recorded by an X-ray beam monitor based on a coded aperture camera that collects radiation scattered from a thin foil placed in the X-ray beam at an oblique angle.

## 1. Introduction

Means to measure X-ray beam shape, size, position and intensity are of paramount importance during both commissioning and routine operation of synchrotron beamlines. Knowledge of beam shape, size and intensity distribution provides valuable information about the performance of the upstream optics. Most beam monitoring devices, both available commercially and developed in-house at synchrotrons around the globe, are concerned with measurements of beam position and total intensity, hence the popular terms of beam position monitor (BPM) and beam intensity monitor (BIM). Typical BPMs and BIMs are capable of providing *in situ* measurements with little effect on the beam. Information about the beam size can be obtained from micro-strip or multi-channel plates ion chambers (Oed, 1988 ▶; Ilinski *et al.*, 2007 ▶) and wire scanners (Fulton *et al.*, 1989 ▶; Ross *et al.*, 1991 ▶; Schmidt *et al.*, 2001 ▶), or by observing X-ray-induced photoluminescence of helium gas (Revesz & White, 2005 ▶). However, it has proved to be a lot more challenging to devise a similarly ‘transparent’ instrument for measuring the shape of the beam cross section, *i.e.* a beam imaging device. The obvious way to obtain beam cross-sectional images is to expose an X-ray camera to the direct beam. Such cameras typically use a phosphor screen, which converts incident X-ray radiation into visible light, optically coupled to a standard CCD or CMOS detector (Bunk *et al.*, 2005 ▶). By using a very thin phosphor screen to reduce beam absorption (Martin *et al.*, 2008 ▶) and designing the camera so that it does not obstruct the beam in full (Fuchs *et al.*, 2007 ▶; Hahn *et al.*, 1998 ▶), a desired configuration for *in situ* measurements can be obtained. However, a very thin phosphor that has small beam absorption results in a low intensity of the visible light recorded by the camera. Moreover, the performance of phosphor screens deteriorates when they are continuously exposed to intense X-ray beams. For white radiation in the hard X-ray range, a transparent beam imaging device based on Bragg reflection from a thin beryllium crystal mounted at 45° relative to the incident beam has been suggested (Fajardo & Ferrer, 1995 ▶). However, the use of such a device as an *in situ* beam monitor is inconvenient in set-ups where the energy of the monochromatic beam changes frequently.

Recently, a new type of X-ray beam monitor based on observations of radiation scattered by a thin film placed in a beam at an oblique angle with a lensless (pinhole) camera has been introduced (van Silfhout *et al.*, 2011 ▶; Kyele & van Silfhout, 2012 ▶). A similar device for measuring the vertical position of the intense hard X-ray beam has also been described elsewhere (Revesz *et al.*, 2010 ▶). The device’s advantages include transparency, longevity, high resolution of beam position measurements and wide operating range of X-ray beam energies and intensities (Kachatkou & van Silfhout, 2013 ▶). In this type of BPM, the beam position is derived from the detected image of the source of scattered radiation. This image is a magnified representation of the beam cross section, which is distorted due to the device geometry and imperfections of its components. In some cases, such as when a coded aperture is used to boost signal levels (Kachatkou & van Silfhout, 2013 ▶), the shape of the detected image differs significantly from the shape of the beam’s cross section. Below, we present a model of the X-ray beam imaging (XBI) process for this new class of BPM device and propose a method to reconstruct cross-sectional images of the incident beam from recorded XBI images. The reconstruction results are compared with the images obtained by exposing an X-ray camera to the direct beam. We also discuss the effect of various device parameters on the quality of reconstructed images.

## 2. Imaging model

**Figure 1:**
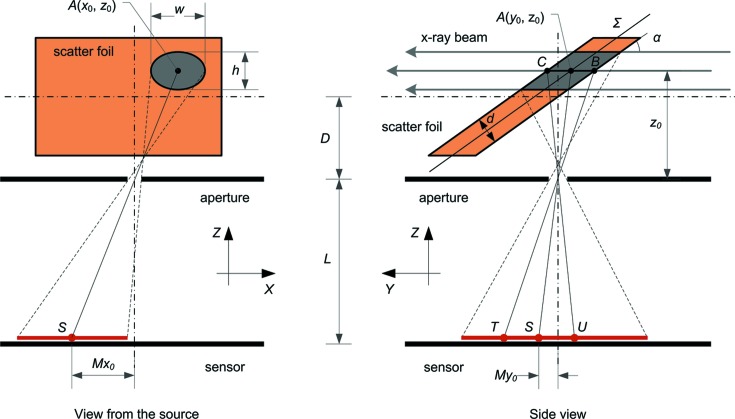
*In situ* X-ray beam imaging geometry (not to scale). A pinhole camera collects the radiation scattered from the scatter foil placed into a beam at an acute angle α. The image recorded by the sensor represents the volume cut in the foil by the beam (dark grey areas).

In the proposed transparent beam monitor, the X-ray beam impinges upon a thin foil made of a low-*Z* material tilted at an angle α with respect to the beam (Fig. 1 ▶) (Kyele & van Silfhout, 2012 ▶; van Silfhout *et al.*, 2011 ▶). Scattered radiation is collected by the aperture of the lensless camera and is recorded by the X-ray sensor. As with the standard pinhole camera, images captured by the sensor are magnified by a factor of *L*/*D*.

Let us consider an imaginary central plane in the middle of the foil, Σ, *i.e.* plane Σ is located so that it is parallel to and equidistant from the foil’s faces. Assume that the origin of the coordinate system defined in Fig. 1 ▶ is at the centre of the aperture. An arbitrary point *A*(*x*_0_, *y*_0_, *z*_0_) of the beam footprint on Σ is projected onto the image plane through the centre of the aperture as point *S* with coordinates (−*Mx*_0_, −*My*_0_), where *M* = *L*/*z*_0_ is the magnification factor. Owing to the foil tilt, coordinates *y*_0_ and *z*_0_ of any point that belongs to Σ are coupled,
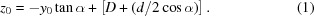
The collection of all points *S*, *s*(*x*, *y*), represents the corresponding projections of all points *A* from the beam footprint on plane Σ. In other words, *s*(*x*, *y*) is the ideal XBI image, an image created by the XBI camera with the infinitesimally thin scatter foil and the ideal, *i.e.* infinitesimally small, pinhole. The relation between the beam cross section (in the *XZ* plane) and this image (in the *XY* plane) is fully described by the magnification factor *M* and equation (1),
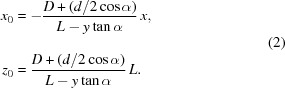
An image *i*(*x*, *y*) created by a practical XBI system is obtained by convolution of image *s*(*x*, *y*) with the XBI impulse response *h*(*x*, *y*),
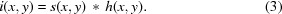
The XBI impulse response is determined by three major factors: the aperture shape and size, the foil thickness and the detector’s point-spread function (PSF). If the X-ray transmission of the aperture is described by *a*(*x*, *y*), then the corresponding contribution to the XBI impulse response is given by *a*[*x*/(*M* + 1), *y*/(*M* + 1)]. Here, the aperture is assumed to be made of a thin foil so that its size and position are the same for radiation scattered from any point of the scatter foil within a practical range of beam positions and sizes. The shape of *a*(*x*, *y*) is not limited by a circle (pinhole) but can represent any pattern, conventionally called a coded aperture, *e.g.* a slit, multiple pinholes or a cross.

To calculate the foil contribution to the XBI impulse response, we consider the case of the infinitesimally thin beam that propagates along the *Y* axis and assume that the XBI aperture is the ideal pinhole. The centre of the beam has coordinates (*x*_0_, *z*_0_) and the intensity distribution across its cross section at the *XZ* plane is given by the Dirac delta function *δ*(*x* – *x*_0_, *z* – *z*_0_). When the beam propagates through the foil, it cuts an infinitesimally thin path *BC* (see Fig. 1 ▶). The projection of *BC* onto the image plane through the pinhole is the segment *UT*. The corresponding intensity distribution is given by
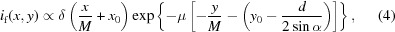
where 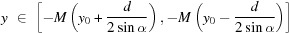
and μ is the linear absorption coefficient of the scattering foil material. The foil impulse response, *h*_f_(*x*, *y*), which describes image blur caused by the finite thickness of the foil, is given by equation (4) for *x*_0_ = 0 and *y*_0_ = 0. The absolute value of *i*_f_(*x*, *y*) and *h*_f_(*x*, *y*) is determined by the dependence of the scattered intensity on the direction of scattering and, therefore, depends on the angle between *BC* and *AS*. However, assuming that the distance between the foil and the pinhole is significantly larger than the thickness of the foil (*D* >> *d*), this dependence is negligible and the proportionality sign in (4) can be replaced by the equality sign if the right-hand side is multiplied by a constant intensity factor. Since this work is focused on the imaging performance of the system and not on performing absolute intensity measurements, the intensity factor is taken to be equal to 1 in the subsequent discussion.

**Figure 2:**
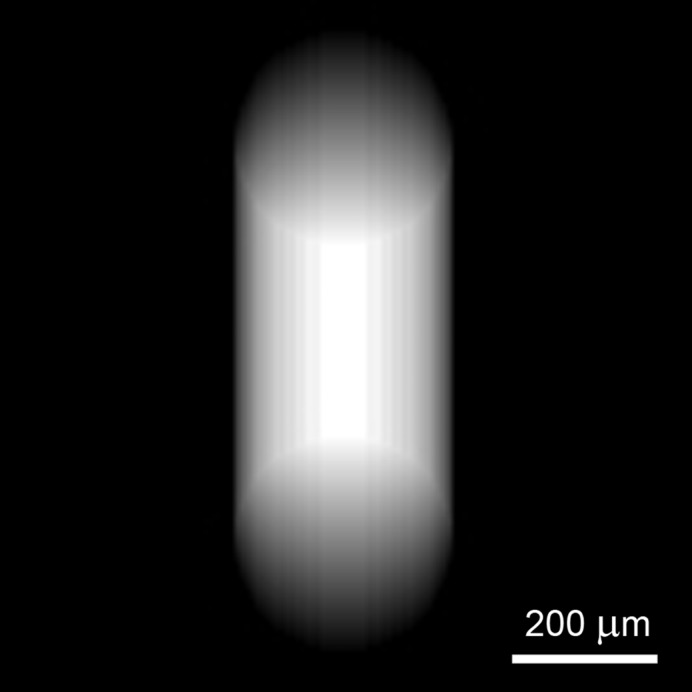
Calculated XBI impulse response for a system equipped with the detector whose PSF is the Dirac delta function; *L*/*D* = 2, α = 27°, 100 µm circular aperture, 125 µm foil with μ = 0.005 mm^−1^.

The combined contribution of the foil and aperture into the XBI impulse response is given by
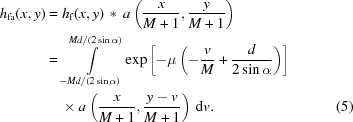
The XBI impulse response *h*(*x*, *y*) is obtained by convolution of *h*_fa_(*x*, *y*) and the detector’s PSF. Note that the magnification factor *M* in (5) depends on *z*_0_ and, consequently, on *y*_0_. Therefore, *h*(*x*, *y*) varies for different locations along the image *Y* direction. However, in practice *M* does not vary much (small beam movements relative to *D*) and, therefore, can be deemed to be constant: *M* = *L*/[*D* + *d*/(2cosα)] ≃ *L*/*D*. For this approximation, the XBI impulse response is spatially invariant. The exponential term in (5), which accounts for the foil thickness, results in the XBI impulse response being elongated along the direction of the foil tilt as demonstrated in Fig. 2 ▶.

## 3. Image reconstruction

**Figure 3:**
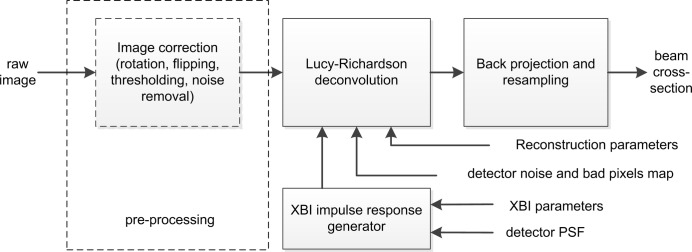
XBI image reconstruction. First, the input raw image undergoes an optional pre-processing step. Then, the image blur introduced by the scattering foil, the aperture and the detector’s PSF is removed by Lucy–Richardson deconvolution with the calculated XBI impulse response. At the final step, geometrical distortions owing to the XBI magnification and the scattering foil tilt are removed by coordinate conversion from the image plane to the beam cross-sectional plane.

To reconstruct the true cross-sectional image of the beam from the recorded XBI image, it is necessary to reverse the effect of each imaging step described in the previous section and apply the corresponding reconstruction routines to the data that are often modified by detector noise and a background signal. A typical XBI image reconstruction pipeline is shown in Fig. 3 ▶.

First, one needs the XBI impulse response which can be calculated *a priori* for a given foil material, the detector and known device dimensions. The detector’s PSF can be obtained from the manufacturer or measured separately (van Silfhout & Kachatkou, 2008 ▶). The effect of the XBI impulse response is then removed by resorting to one of the well established deconvolution techniques (Jansson, 2012 ▶). In this work we use the Lucy–Richardson algorithm (Hanisch *et al.*, 2012 ▶).

When reconstructing images obtained from a practical system, apart from the XBI impulse response one also needs to factor in the effect of detector noise. The detector noise primarily consists of two components: photon-counting noise and readout noise. The photon-counting noise has a Poisson distribution and is implicitly accounted for in the derivation of the Lucy–Richardson iteration (Hanisch *et al.*, 2012 ▶). The readout noise is typically described as additive noise with a Gaussian distribution. As shown by Hanisch *et al.* (2012 ▶), the Lucy–Richardson algorithm can be modified to accommodate this type of noise. The magnitude of the readout noise required for the modified Lucy–Richardson iteration is approximated by the variance of an unexposed image taken with the same detector and under the same conditions as subsequent XBI images. Other types of noise such as impulse noise, bad pixels and background signal (pixel dark current, X-ray air scattering *etc.*) can also be accounted for during the Lucy–Richardson iteration (Hanisch *et al.*, 2012 ▶). However, we find that it is sometimes more convenient and efficient to correct for these distortions during the pre-processing step using pixel interpolation and dedicated filters such as median and threshold filters. Also, during the pre-processing step, the orientation of XBI images can be corrected in order to coincide with the orientation of the corresponding XBI impulse response.

The result of the Lucy–Richardson deconvolution is the estimation of *s*(*x*, *y*) in equation (3) which, in order to produce the desired beam cross-sectional image, needs to be projected back onto the *XZ* plane using equations (2). The values of *s*(*x*, *y*) are typically represented on a rectangular grid formed by the detector’s pixels. Equations (2) deform this grid. Therefore, for convenience, the reconstructed cross-sectional image needs to be resampled using a new rectangular grid. The intensity values corresponding to the new grid locations are computed from the original deformed grid by a suitable interpolation algorithm (*e.g.* bilinear interpolation). The combination of back-projection and resampling forms the last XBI image reconstruction stage in Fig. 3 ▶.

## 4. Experimental results

The performance of the XBI system was tested in experiments with both unfocused and focused X-ray beams of various sizes as produced by bending-magnet beamlines, and using both a pinhole and a coded aperture XBI set-up for comparison.

**Figure 4:**
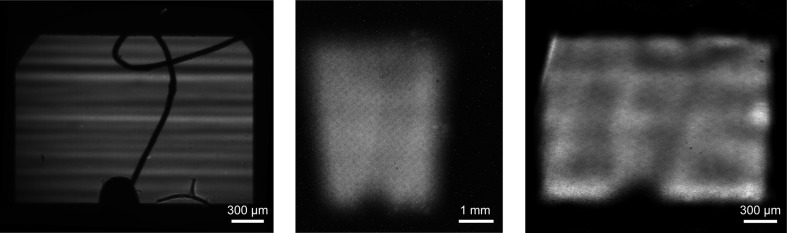
Images of a 50 µm-thick gold wire placed into an unfocused monochromatic (15 keV) beam at bending-magnet beamline B16 (DLS, UK): direct image taken with an X-ray microscope (left); raw XBI image obtained using 400 µm circular aperture and *L*/*D* = 2 (centre); beam cross-section image reconstructed using 20 Lucy–Richardson iterations (right). Other XBI parameters: 125 µm Kapton foil, α = 27.8°, and *L* = 5 mm, *D* = 10 mm, CMOS detector with 7 µm pixels fibre-optically coupled with a Gd_2_O_2_S:Tb scintillator foil (van Silfhout & Kachatkou, 2008 ▶), integration time 3 s.

In the first experiment we collected images from an unfocused 15 keV beam at beamline B16 at the Diamond Light Source (DLS, UK) (Fig. 4 ▶). The top-hat beam was shaped by slits opened up to approximately 2 mm × 1.5 mm (h × v). A feature consisting of a 50 µm-thick gold wire glued to the tip of a board pin was deliberately put into the beam. A high-resolution direct image of the beam taken with the X-ray microscope equipped with a 10× objective lens and a pco.4000 CCD camera (Sensitive Cameras, 2008 ▶) clearly shows the tip of the pin, the wire and also the variations in the beam intensity owing to the multi-layer monochromator (horizontal stripes). The microscope was installed downstream from the XBI device. The XBI device was set up with a 400 µm circular aperture (tungsten), 125 µm Kapton foil and with *L*/*D* = 2. An unprocessed XBI image shown in Fig. 4 ▶ hints at the presence of the pin’s tip in the beam, but the details of both the wire and the striped intensity variations are hidden from view. The tapered appearance of the XBI image is a result of the spatially varying XBI magnification caused by the foil tilt. The long side of the image is parallel to the *Y* axis (Fig. 1 ▶) and the beam propagates in the direction from the top to the bottom of the image so that the pixels at the top of the image register the radiation scattered from the part of the foil closest to the aperture plane, and *vice versa*. As a result, the top part of the image has a higher magnification than the bottom one. Note that in the reconstructed beam cross-sectional image in Fig. 4 ▶ the effect of the spatially varying XBI magnification is removed. This image, which was obtained using 20 Lucy–Richardson iterations followed by the back-projection and resampling as described in the previous section, clearly identifies the presence of the wire.

**Figure 5:**
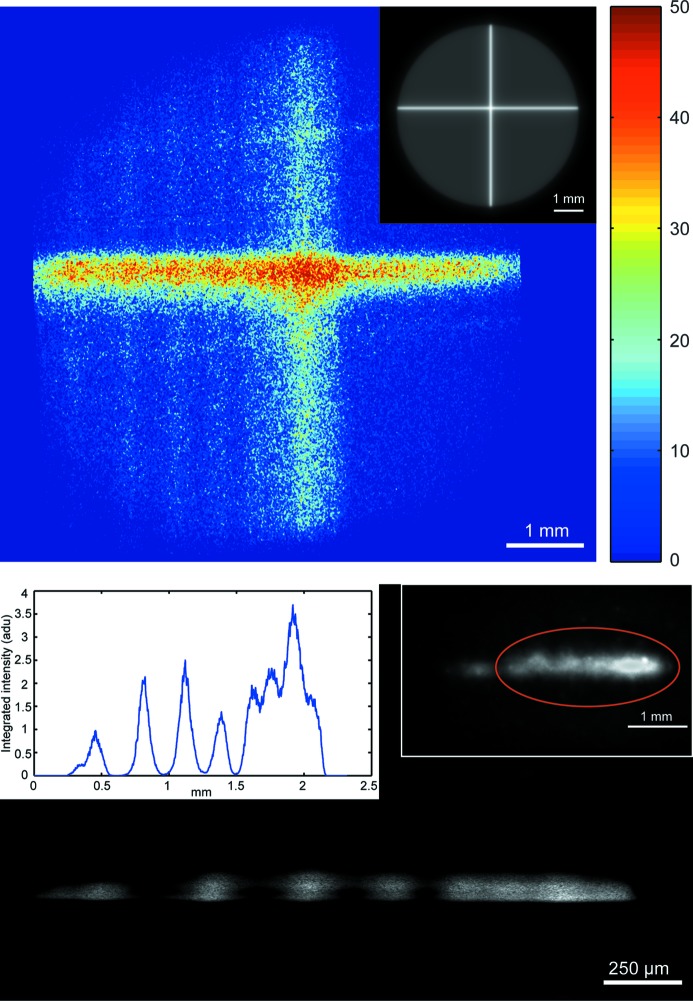
Images of focused beam (12.7 keV) at bending-magnet beamline BM26A (ESRF, France) taken using a cross-shaped aperture (formed by 3000 µm × 25 µm slits laser cut in a 13 µm-thick stainless steel foil) suspended on a 0.2 mm-thick molybdenum foil with a hole of about 2 mm in diameter. Top: the XBI image obtained by averaging ten raw images corrected by background subtraction and the calculated XBI impulse response (inset). Bottom: the reconstructed cross-sectional image with the corresponding horizontal image profile (left inset) and the image obtained by exposing the X-ray camera directly to the beam (right inset). XBI parameters: *L*/*D* = 2, 50 µm copper foil, α = 18.7°, CMOS detector with 7 µm pixels fibre-optically coupled with a Gd_2_O_2_S:Tb scintillator foil (van Silfhout & Kachatkou, 2008 ▶), integration time 0.25 s.

In the second experiment we have replaced the pinhole aperture plate by a coded aperture. As shown by Kachatkou & van Silfhout (2013 ▶), cross-shaped apertures enable a better signal-to-noise ratio (SNR) in beam position measurements and it is therefore important to understand the implications of their usage for beam imaging. To investigate the performance of the cross-shaped coded aperture we have performed experiments with a focused monochromatic (12.7 keV) beam produced by a double Si(111) crystal monochromator at the BM26A beamline at the European Synchrotron Radiation Facility (DUBBLE CRG, ESRF, France) using an aperture formed by two 3 mm-long and 25 µm-wide slits laser-etched in a 13 µm-thick stainless steel sheet. This relatively thin sheet was chosen to adhere to the thin aperture approximation (see §2). The cross-shaped aperture was then suspended on a 0.2 mm-thick molybdenum foil with a 2 mm-diameter hole. The average of ten unprocessed XBI images and the corresponding calculated XBI impulse response are shown in Fig. 5 ▶. The large grey circle circumscribing the cross in the XBI impulse response image reflects the fact that the stainless steel sheet was too thin to entirely prevent the scattered radiation from reaching the sensor. The reconstructed cross-sectional image in Fig. 5 ▶ contains detailed information about the side lobes caused by a ribbed sagittally focusing monochromator, which has not been curved properly. This image is compared with the direct image of the same beam taken by focusing a standard CMOS camera on a scintillator screen placed in the beam behind the XBI system. Although the cross-sectional image obtained with the XBI system is not large enough to include the whole beam cross section, it does not suffer from saturation and provides a more detailed view of the bright part of the beam thanks to the XBI magnification.

**Figure 6:**
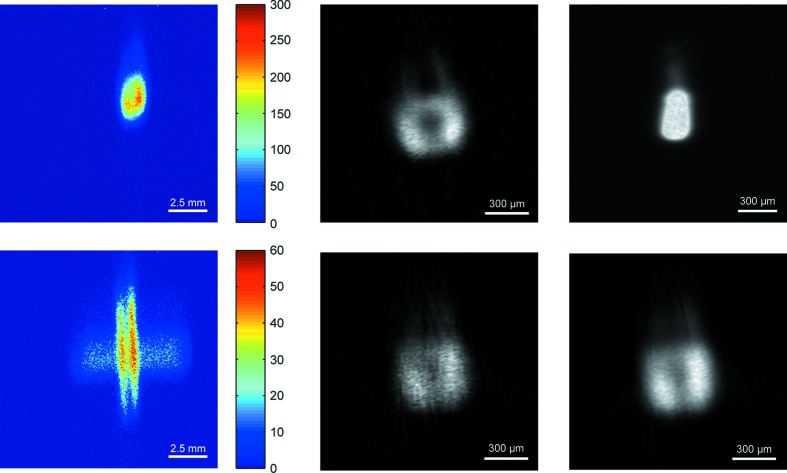
Images of a focused monochromatic (12 keV) beam as measured at bending-magnet beamline B16 (DLS, UK). Top row: a raw unprocessed XBI image obtained using a 200 µm circular aperture, *L*/*D* = 3 and counting time of 0.5 s (left); corresponding reconstructed beam cross-section image (centre); reference direct image taken with an X-ray Eye camera set up 20 cm downstream and near the focal point (right). Bottom row: a raw XBI image obtained using a cross aperture with slit size of 1700 µm × 25 µm, *L*/*D* = 3 and counting time of 1 s (left); corresponding reconstructed beam cross-sectional image (centre); beam cross-sectional image reconstructed from an image obtained by averaging ten raw XBI images (right). Beam cross-sectional images were reconstructed using 16 Lucy–Richardson iterations. Other XBI parameters: 125 µm Kapton foil, α = 25°, noiseless Medipix-2 detector with 55 µm pixels (Llopart *et al.*, 2002 ▶).

Finally, in Fig. 6 ▶ we compare the XBI images of a 12 keV X-ray beam focused with a torroidal mirror that were obtained using both circular and cross-shaped apertures at B16 (DLS, UK). The reference image in Fig. 6 ▶ was recorded by an X-ray Eye camera (Photonic Science X-ray MiniFDI; Photonic Science, 2013 ▶) installed near the focal point. The XBI system was set up at about 20 cm upstream from the X-ray Eye. The XBI images suggest that before the focal point the beam is split into two symmetrical parts indicating a problem with the torroidal mirror. A relatively large circular aperture (200 µm diameter, tungsten) delivers a good SNR; however, the reconstructed image has a rounder shape than in the reference image. The raw XBI images obtained using a tungsten cross-shaped aperture with 1.7 mm × 25 µm slit size have a significantly lower SNR even though the detector counting time was twice as long as for the circular aperture. As a result, image artefacts started to appear in the reconstructed images even after a few Lucy–Richardson iterations (Fig. 6 ▶, centre bottom image). However, by averaging ten raw images these artefacts were completely removed and the shape of the left and right parts of the split beam in the final image matches the shape of the beam in the reference image (Fig. 6 ▶, right bottom image).

## 5. Discussions

The imaging resolution of a pinhole camera system is determined by the size of its aperture. The optimal size for the aperture can be estimated using the following equation, which was initially derived by Petzval and later modified by Rayleigh (Mielenz, 1999 ▶),
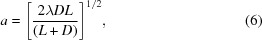
where λ is the wavelength of the light and *a* is the aperture diameter. For 12.7 keV (λ ≃ 0.98 Å) and 15 keV (λ ≃ 0.83 Å) X-rays used in this work, the corresponding optimal pinhole diameters are 0.86 µm and 0.74 µm, respectively. Such small pinholes cannot be used in practice because their transmission is too low to obtain images with a reasonable SNR without resorting to very long integration times. The typical use of the XBI system is for beam position monitoring for which a good SNR and fast frame rates are of paramount importance. Therefore, one is often forced to use pinholes with sizes significantly larger than the optimum value. This leads to residual blur and reconstruction artefacts in the beam cross-sectional images (see Figs. 4 ▶ and 6 ▶). In earlier work (Kachatkou & van Silfhout, 2013 ▶) we have shown that cross-shaped coded apertures increase the resolution of beam position measurements by improving the SNR of the measured image profiles. Moreover, the resolution of beam position measurements can be increased even when using the cross aperture with the width of slits considerably smaller than the diameter of the pinhole. We argue that in this case the quality of reconstructed beam cross-sectional images will also improve. Narrow slits ensure that the effective size of the aperture in vertical and horizontal directions of the image is closer to the optimum predicted by equation (6), which results in more precise image reconstruction (Fig. 6 ▶). However, for artefact-free deconvolution, the whole cross-shaped XBI image should fit the light-sensitive area of the detector. Therefore, the length of the slits forming the cross should be carefully chosen so that this condition is satisfied in all practical positions of the beam, and, at the same time, the SNR of image profiles is sufficiently high to attain the required resolution of beam position measurements.

The imaging resolution of the XBI system is greatly influenced by the scatter foil. A thicker foil causes blurring of the reconstructed images whereas a thin foil scatters very few X-rays resulting in a low SNR and, consequently, a poor quality of reconstructed beam images. As a result, the foil choice is always a compromise between the image and profile SNR and image blur. Using a thin layer of a high-*Z* material as the scatter foil would address the blurring of the recorded images whilst keeping the recorded SNR sufficiently high.

Naturally, the SNR of the XBI images and, consequently, the quality of reconstructed beam cross-sectional images is determined by the intensity of the incident X-ray beam. Our experiments demonstrated that even at bending-magnet beamlines the XBI device is capable of providing a beam cross-sectional image at least once every second. At insertion-device beamlines that produce up to two orders of magnitude higher flux, XBI images of significantly higher quality can be achieved with acquisition times reduced by a factor of ten.

Although we have not included absolute intensity measurements in our derivation of the impulse response of the XBI device, it will come as no surprise that our measurement method is suitable for recording the beam intensity of the incident monochromatic beam in photons per second. The scattering yield of Kapton as a function of energy has been published elsewhere (Kachatkou & van Silfhout, 2013 ▶; Zontone, 2012 ▶).

Owing to the XBI geometry, the device is capable of recording magnified images of the incident beam. Recently, we have recorded images of a highly focused beam of 5 µm r.m.s. in an experiment which used compound refractive lenses (Kachatkou *et al.*, 2013 ▶).

The image formation model described in this work can be used to estimate the Gaussian width of image profiles required for evaluating the spatial resolution of beam position measurements as described by Kachatkou & van Silfhout (2013 ▶). Firstly, an XBI image is computed by projecting the expected X-ray beam cross section onto the image plane using equations (2) and convolving the corresponding projected image with the XBI impulse response. Secondly, the image profiles are calculated by summing XBI image rows and columns and fitting with a Gaussian function to obtain the respective Gaussian width values. The system noise can also be added to the model if necessary. This method will provide a much more precise estimate of image profiles’ Gaussian width than the empirical approach used by Kachatkou & van Silfhout (2013 ▶).

The computational cost of the XBI image reconstruction method is dominated by the complexity of the Lucy–Richardson deconvolution combined with the back-projection and resampling step. Lucy–Richardson deconvolution requires several arithmetical operations performed on all image pixels and four fast Fourier transforms per iteration (Hanisch *et al.*, 2012 ▶). The back-projection and resampling of the image can be implemented by computing the intensity of each pixel of the beam cross section as a bilinear interpolation of intensity values of pixels adjacent to the location of the projection of the target pixel to the image plane. The interpolation coefficients are constant and need to be evaluated only once for all images taken with a given XBI set-up. Image reconstruction presented in this work was performed in MathWorks MATLAB R2012a on a Dell OptiPlex 745 desktop computer equipped with an Intel Core 2 6600 dual core processor and 4 GB of RAM. The Lucy–Richardson deconvolution and back-projection steps for Fig. 4 ▶ (1066 × 979 pixels raw image) took approximately 19 s whereas for Fig. 6 ▶ only 0.3 s was required (256 × 256 pixels raw image). The data processing speed can be significantly improved by parallelizing and implementing the reconstruction algorithms on dedicated hardware such as digital signal processors (DSPs), reconfigurable logic (FPGAs), application-specific integrated circuits (ASICs), or general purpose graphics processing units (GPGPUs). A dedicated embedded image processing system pipelined with the image acquisition electronics would be an ideal solution to provide real-time images of the X-ray beam cross section.

## 6. Conclusion

This work introduces a detailed model of the beam imaging process taking place in the recently presented XBI/BPM device based on imaging X-ray radiation scattered from a thin foil of a low-*Z* material with a lensless camera. Using this model, a reconstruction method to obtain beam cross-sectional images from XBI raw data was developed. The results of the experiments with synchrotron radiation prove the suitability of the presented imaging method for *in situ* X-ray beam characterization and demonstrate the advantages of having a beam monitor capable of providing live images for identifying problems with X-ray optics. In cases of sufficiently high intensity of the incident X-rays, real-time beam imaging can be achieved by implementing the reconstruction method in a dedicated image processing hardware. The presented XBI model also complements the calculations of beam position measurements resolution described elsewhere (Kachatkou & van Silfhout, 2013 ▶) by providing an estimate of the width of XBI image profiles.
